# Improving the diversity of the *PLOS ONE* editorial board

**DOI:** 10.1371/journal.pone.0308492

**Published:** 2024-08-26

**Authors:** Shein Ei Cho, Emily J. Chenette

**Affiliations:** Public Library of Science (PLOS), San Francisco, California, United States of America; Public Library of Science, UNITED KINGDOM OF GREAT BRITAIN AND NORTHERN IRELAND

At PLOS, Open Science is part of our DNA. Truly Open Science is more than just ensuring that knowledge is open and accessible: it also requires that “the production of that knowledge itself is inclusive, equitable and sustainable” [1]. We recognize that PLOS has an important role in driving inclusivity and equity in research. Indeed, several years ago, we reaffirmed our commitment “to ensur[ing] that our journals are representative of broader society as well as the academic community, and that the voices of different communities or groups of researchers are heard and play an integral role in our journals” [2]. We are therefore excited to share our recent progress and future plans to support a more geographically diverse editorial board.

Structural inequality is a well-documented problem in academia, leading to a striking lack of diversity in senior roles in our community [3–5]. For better or worse, hiring, tenure and promotion decisions are overwhelmingly made on the basis of a candidate’s publication record–though at PLOS we think that this is much too narrow a lens by which to view productivity [6]. It therefore follows that one way to improve diversity in senior positions is to ensure that minority and historically excluded researchers have equitable access to publishing options. Others have found that a diverse editorial board correlates with the diversity of scholarship published in the journal [7], so maintaining an editorial board that reflects the composition of the research community can help ensure that researchers have truly equitable opportunities for promotion and advancement.

Our commitment to ensuring the diversity of our editorial board was not made solely to help redress inequities in publishing, however. To effectively serve its communities, a journal needs to be shaped and managed by members of those communities. Indeed, a recent paper has shown that in the field of race scholarship, a diverse board correlated with a more positive perception of the journal [8]. Furthermore, a 2018 survey of members of the Committee on Publication Ethics (COPE) found that editors and publishers recognise the importance of diversity in the peer-review process, suggesting that efforts to increase diversity “could potentially both improve peer review quality and reduce bias” [9].

At *PLOS ONE*, we also rely on the diverse voices and opinions of our Editorial Board Members in shaping editorial policy. For example, before launching our policy on inclusivity in global research, we consulted extensively with members of our community to understand their concerns around helicopter research and to co-develop a policy that addressed these issues [10]. Our internal data also suggests that *PLOS ONE* Academic Editors are more likely to agree to handle papers from authors in their same geographical regions, likely supporting a more rapid peer-review process. Thus, we must ensure that the Editorial Board reflects the geographical diversity of *PLOS ONE* authors to support efficient peer review, bolster the positive perception and trust in published papers, and ensure that the journal continues to meet the needs of our diverse community.

**Fig 1:**
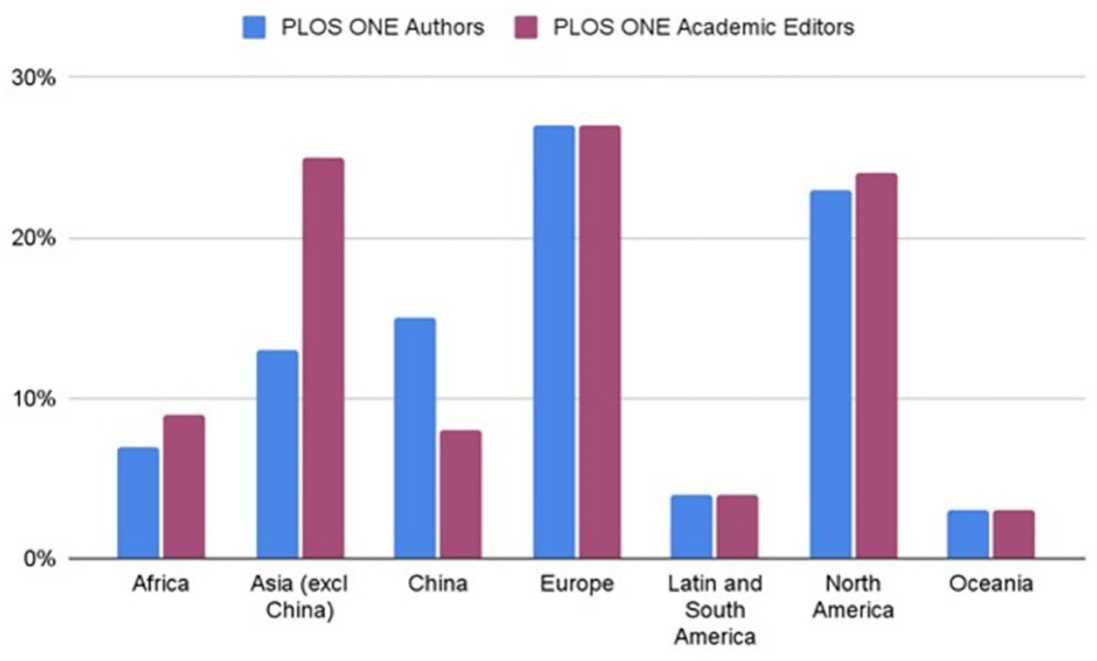
Geographical diversity of published *PLOS ONE* corresponding authors in 2023 compared to the geographical diversity of the *PLOS ONE* editorial board in December 2023.

Although structural inequality has many facets, our efforts thus far have focused on improving the geographic diversity of the *PLOS ONE* editorial board. As a first step, we assessed the geographical diversity of corresponding authors who published with *PLOS ONE*, as well as the composition of the *PLOS ONE* editorial board (Fig 1). When looking at data from 2023, we found that the proportion of authors and editors from Africa, Europe, Latin and South America, North America and Oceania were similar (if not identical). However, the proportion of authors from mainland China is far higher than the number of Academic Editors from the region; conversely, the proportion of Academic Editors from other countries in Asia is far greater than the proportion of corresponding authors.

To address this discrepancy, we are actively recruiting new Academic Editors from institutions in China, with a particular focus on research areas where submissions from China are the highest. In addition, we are stepping up our outreach efforts in Asia to increase the number of papers that we publish from the region. Each quarter, we re-analyze the geographical diversity of our Academic Editors and corresponding authors, and adjust recruitment plans for the next three months as needed. These efforts have begun to bear fruit, as the proportion of Academic Editors from China has increased by a percentage point in the first two quarters of this year.

Improving geographic diversity is an important step towards achieving our aim of having a more representative and inclusive editorial board, but there is more that we need to do to address other inequalities on our board. At present, we do not have insight into other parameters of diversity, limiting our ability to understand and eliminate other potential sources of bias. We are also mindful of the need to ensure that *PLOS ONE* is representative of its entire community, and not just of the authors who choose to publish with us. Understanding the composition of the research ecosystem within which *PLOS ONE* sits will be an important future endeavor.

While we consider how best to address these issues, we have taken additional steps to support diversity and inclusivity in peer review. We have added a statement to all PLOS journal websites to remind ourselves and our community of our commitment to building diverse editorial boards, and to encourage candidates from diverse backgrounds to apply to join the board. The full statement is: “The PLOS Editorial Boards are a diverse, worldwide panel of expert researchers. We are committed to building and promoting an inclusive community that welcomes diversity in age, gender identity, race, sexual orientation, physical or mental ability, ethnicity, religion, or belief.” We are also providing additional support to our board members to foster a more inclusive journal community. As an example, we recently published a Diversity, Equity and Inclusion Guide that lists actionable items that our Academic Editors can do to promote a diversity of viewpoints in the peer-review process.

We are also redoubling efforts to meet with our Academic Editors and Section Editors in person to discuss challenges relating to scholarly publishing and understand how we can work with them to improve opportunities at *PLOS ONE* for early career researchers and underrepresented groups. Journal staff have received additional training in identifying and recruiting potential Academic Editors from more diverse sources. Finally, we participate in a number of industry initiatives to promote diversity, equity and inclusivity in scholarly publishing. Through these steps, we can make meaningful improvements to the diversity of our editorial board, and continue to hold ourselves accountable to supporting an equitable, participatory open science future.
